# Navigating Complexity in Pediatric NMOSD: Unusual Symptoms and Adverse Reactions: A Case Report

**DOI:** 10.3390/reports8010006

**Published:** 2025-01-08

**Authors:** Oana-Aurelia Vladâcenco, Radu-Ștefan Perjoc, Eugenia Roza, Raluca Ioana Teleanu

**Affiliations:** 1 Clinical Neuroscience Department, Pediatric Neurology, Faculty of Medicine, “Carol Davila” University of Medicine and Pharmacy Bucharest, 020021 Bucharest, Romania; oana-aurelia.vladacenco@drd.umfcd.ro (O.-A.V.); eugenia.roza@umfcd.ro (E.R.); raluca.teleanu@umfcd.ro (R.I.T.); 2 “Dr. Victor Gomoiu” Children’s Hospital, 022102 Bucharest, Romania

**Keywords:** NMOSD, paediatric, demyelinating disorder, AQP4 antibodies

## Abstract

**Background and Clinical Significance**: Neuromyelitis optica spectrum disorder (NMOSD) is a rare autoimmune demyelinating disorder of the central nervous system, characterized by the presence of aquaporin-4 (AQP4) antibodies and a high relapse rate. We provide information about the diagnosis, unusual symptoms, and treatment of a paediatric patient with NMOSD. **Case Presentation**: A 14-year-old girl was hospitalized for weakness and paraesthesia of the lower limbs (LL). The patient underwent detailed investigations and was diagnosed with NMOSD and cryptogenic organizing pneumonia. Initial treatment with methylprednisolone and prednisone yielded a favourable response. Therapy with mycophenolate was initiated. However, the patient experienced two more relapses, prompting the use of rituximab therapy with a favourable outcome and a two-year relapse-free follow-up period. **Conclusions:** Patients with NMOSD may have multisystemic inflammation, including organs outside the central nervous system. Our case report highlights a case of NMOSD, pulmonary involvement, and unusual adverse reactions to rituximab.

## 1. Introduction and Clinical Significance

Neuromyelitis optica spectrum disorder (NMOSD) is a rare inflammatory demyelinating disorder of the CNS, with a typical clinical presentation of recurrent attacks of optic neuritis and acute transverse myelitis. However, in 2015, the International Panel for NMO Diagnosis (IPND) revised the diagnostic criteria and expanded the core clinical characteristics to include area postrema syndrome, acute brainstem syndrome, symptomatic narcolepsy or acute diencephalic syndrome, and symptomatic cerebral syndrome [1]. Paediatric-onset cases are rare, accounting for only 3–5% of all NMOSD cases [2].

AQP4 antibodies (AQP4-ab) are detected in the majority of patients with NMOSD and play a crucial role both in the pathogenesis and diagnosis [1]. These IgG antibodies bind to the AQP4 channels on astrocytes, leading to the activation of the complement cascade. This process damages astrocytes and, in turn, affects oligodendrocytes, resulting in demyelination and neuronal loss [3,4]. These advances in the understanding of the pathophysiology of NMOSD have led to development and approval of new targeted treatments. In recent years, several treatments have been approved for patients with AQP4-positive NMOSD, including satralizumab, which is also approved for pediatric patients [5]. However, rituximab, a drug commonly used for NMOSD, was approved exclusively in Japan in June 2022 and remains off-label in other regions. While central nervous system disorder is the main hallmark of the disease, the involvement of systems such as muscular and pulmonary systems has been described in rare case reports [6,7].

This report aims to describe a complex case of pediatric NMOSD with pulmonary involvement characterized by cryptogenic organizing pneumonia (COP), along with an unusual adverse reaction to rituximab treatment as a hallmark feature. While several cases of NMOSD with pulmonary involvement have been reported, a clear link between the two remains uncertain. A better understanding of this relationship could improve the management of such patients.

## 2. Case Presentation

**Figure 1:**
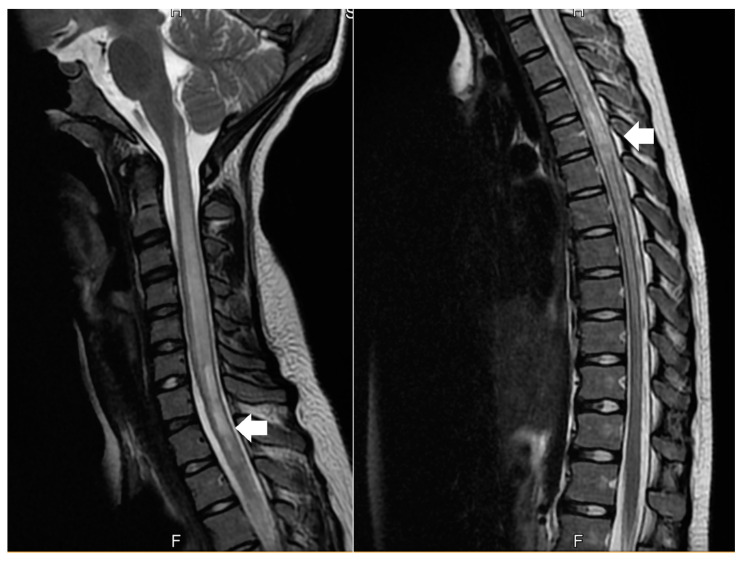
Spinal MRI showing cervical and thoracic (C6-T8) hyperintense lesions on T2-weighted imaging (White arrows).

We report a case of a 14-year-old female presenting with weakness and paraesthesia of the lower extremities and urinary retention with bladder globe. Her recent history revealed interscapular pain and urinary difficulties, one week prior to the onset of weakness. The neurological exam revealed asymmetric lower limb weakness (Medical Research Council, grade 4 left, grade 3 right), asymmetric deep tendon reflexes (diminished on the right side, grade 2—The National Institute of Neurological Disorders and Stroke Muscle Stretch Reflex Scale) and paraesthesia. Serological tests for hepatitis A, hepatitis B, syphilis, HIV, Epstein–Barr virus, Cytomegalovirus, and systemic lupus erythematosus were negative. Serum VDRL was also negative. Anti-MOG antibodies were negative. CSF studies were unremarkable. Serum AQP4 antibodies were detected using the enzyme-linked immunosorbent assay (ELISA) method, with a titer of <1/1000. MRI of the spine showed T2 hyperintense lesions in the cervical and thoracic regions (C6-T8) (Figure 1), with mild spinal cord expansion.

**Figure 2:**
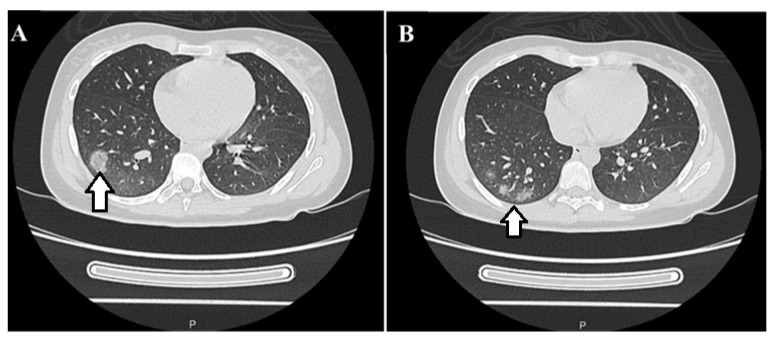
(**A**,**B**) CT scan of the chest showing ground opacities and patchy consolidation in the periphery of the right lower (white arrows) lobe before methylprednisolone.

The MRI also visualized a T2 hyperintense lesion in the right lower pulmonary lobe. A computed tomography scan of the chest confirmed the right lower lobe consolidation suggestive of COP (Figure 2).

**Figure 3:**
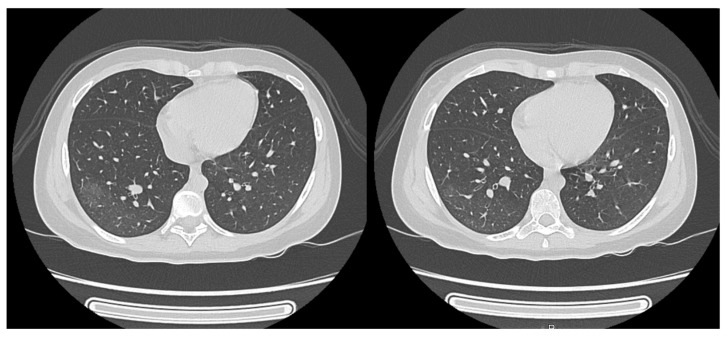
CT scan of the chest after treatment with methylprednisolone.

**Figure 4:**
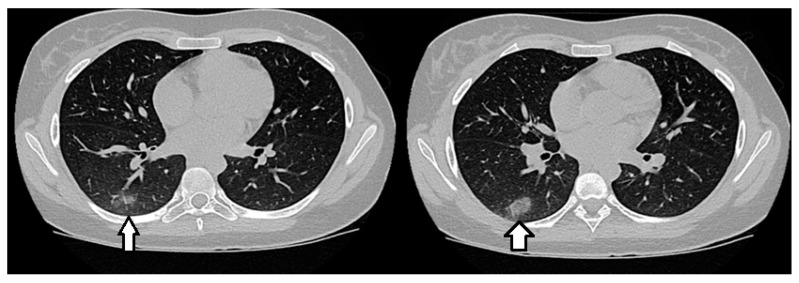
CT scans of the chest during the first relapse, showing ground opacities (white arrows) in the right lower lobe.

The patient had no respiratory symptoms, and the pulmonary examination was unremarkable. Extensive investigations, including the rapid antigen test for SARS-CoV-2 and Influenza A/B, a serological test for SASRS-CoV-2, and Mycoplasma pneumoniae, were negative. Following consultation with a pulmonologist, an infectious disease specialist ruled out infectious causes, including tuberculosis, as well as sarcoidosis, leading to a diagnosis of COP. A diagnosis of NMOSD was established based on the 2015 IPND criteria [1] and the patient was prescribed pulse therapy with methylprednisolone (30 mg/kg/day for 5 days), followed by a tapered course of oral prednisone. Following glucocorticoid treatment and physical therapy, she experienced complete remission of the lower limb weakness but did continue to present bilateral Babinski signs, the feeling of incomplete bladder emptying, and urinary retention. A follow-up chest CT scan showed the resolution of COP (Figure 3). Considering the diagnosis of NMOSD, the patient was started on maintenance therapy for attack prevention with 1 g/day of mycophenolate. Five months after the initial attack, the patient developed painful tonic spasms, which were effectively managed with carbamazepine at a dose of 450 mg/day. Six months after the first attack, the patient experienced another relapse with lower limb weakness (Medical Research Council grade 3 on the left and grade 3 on the right). MRI of the spine revealed lesions characteristic of acute myelitis, as well as a T2 hyperintense lesion in the right pulmonary lobe. A chest CT showed the development of new nodules (Figure 4). Notably, the patient did not exhibit any respiratory symptoms.

Despite treatment with mycophenolate, the patient experienced two additional relapses, prompting the initiation of a rituximab course at 375 mg/m^2^ weekly for four doses. Seven days after the second rituximab infusion, the patient developed severe neutropenia (0.31 × 10^3^/μL), scalp oedema, a malar rash, and prolonged febrile syndrome. Her urinalysis and blood cultures were negative, but Candida spp. was identified in her stool samples and treated accordingly. A skin biopsy from the edematous region revealed non-specific inflammatory changes. As a result, mycophenolate was discontinued 13 months after initiation. Due to the patient’s significant adverse reactions following the second rituximab infusion, the treatment course was stopped, and her CD19 levels were monitored regularly (Table 1). At 12 months following the initial rituximab course, the patient remained relapse-free, with a CD19 level of 8.7%. However, given the adverse reactions caused by the second infusion, the family chose to delay the third dose. At 18 months, the patient’s CD19 level was 7%, and she received another rituximab infusion at 375 mg/m^2^ without any adverse reactions. At the last follow-up, 30 months after the initiation of rituximab, the patient is relapse free.

## 3. Discussion

Our report highlights a complex paediatric case of NMOSD with unusual lung manifestation, painful tonic spasms, and an unexpected adverse reaction to rituximab (scalp oedema).

Paediatric NMOSD is a rare demyelinating disorder associated with antibodies targeting AQP4, a water channel found abundantly in the spinal cord grey matter, periaqueductal, and periventricular regions. However, AQP4 is also known to be expressed in the nasopharyngeal, tracheal, and bronchial epithelium, possibly explaining the association between NMOSD and COP [8,9]. Several cases have been reported in elderly patients and one case has been reported in the paediatric population [7,10,11]. Our patient exhibited the first episode of COP associated with the onset of NMOSD, without any respiratory symptoms. Nevertheless, during her first NMOSD relapse, new pulmonary lesions were observed. Notably, the patient received corticosteroids and mycophenolate, treatments commonly used for COP [12]. However, as these were used for the treatment and prevention of NMOSD attacks, the duration of steroid therapy was shorter than what is typically prescribed for COP. Since no CT scans were performed between the two NMOSD attacks due to concerns about cumulative radiation exposure, it remains uncertain whether the new lesion appeared during steroid tapering or concurrently with the NMOSD relapse. Considering the number of CT scans performed in a short period of time and the lack of respiratory symptoms, we did not conduct a follow-up CT. Nonetheless, subsequent spinal MRI did not reveal any pulmonary lesions.

NMOSD attacks are treated initially with high-dose methylprednisolone. Preventive treatment is essential to reduce the risk of relapses and new disabilities. Several treatment options, such as azathioprine, mycophenolate mofetil, rituximab, and satralizumab, are available for paediatric patients. However, most are used off-label, with only satralizumab approved for NMOSD patients aged 12 years and older [5]. Data on paediatric patients remain limited, and there is no consensus regarding the first-choice treatment [13,14]. However, therapeutic antibodies such as rituximab and satralizumab demonstrate better efficacy compared to classical immunosuppressants [15,16,17,18]. Nonetheless, these data are mostly derived from studies conducted in adult populations. Several factors should be considered when selecting a treatment (e.g., age, comorbidities, family planning, and patient preferences). Furthermore, the optimal doses of rituximab have yet to be determined, and several dosing options are currently in use: 375 mg/m^2^/ once weekly for 4 weeks; 500 mg/m^2^/ dose (max 1 g) two infusions 2 weeks apart; redosing regimens of 500–750 mg/m^2^ when CD19 > 1% or every 6 months. In our case, we chose a regimen of 375 mg/m^2^ administered once weekly for four weeks. The treatment was discontinued after two doses due to adverse reactions. At 18 months after the initial rituximab regimen, the patient received an additional dose in response to a CD19 rebound, this time without any adverse reactions. While neutropenia, skin rash, and fungal infections are recognized adverse effects of rituximab, to the best of our knowledge, scalp oedema has not been previously reported. However, a variety of dermatological adverse reactions, including skin rash, pruritus, epidermolysis bullosa, and Stevens–Johnson syndrome, have been documented. Considering the timing of scalp oedema development, its remission without specific treatment, and the nonspecific inflammation described in the skin biopsy, we propose scalp oedema as a possible adverse reaction to rituximab treatment. Nonetheless, no clear correlation can be established.

Several limitations are present in this study. First, as this is a case report, the findings cannot be generalized, and a cause–effect relationship cannot be established. Regarding pulmonary involvement, due to the cumulative dose of irradiation, we were unable to perform a follow-up CT scan after the second findings suggestive of COP. Furthermore, we did not perform a lung biopsy, and AQP4 staining was not possible.

## 4. Conclusions

Patients with NMOSD may experience multisystemic inflammation, affecting organs outside the central nervous system. Although rare case reports of NMOSD associated with COP have been described, the currently available data are limited, especially in children, with only one other paediatric case reported. Our case adds to the current literature by providing a clear description of a paediatric case of organizing pneumonia present at the onset of NMOSD, and a possible COP relapse during the second attack of NMOSD. Organizing pneumonia might represent a systemic manifestation of NMOSD secondary AQP4-ab targeting pulmonary tissue. Therefore, when facing a patient with a possible diagnosis of NMOSD or a patient with relapse, a careful pulmonary evaluation should be performed in order to provide adequate management. Furthermore, we describe a possible adverse reaction to rituximab, which, to our knowledge, has not been previously reported.

## 5. Patient Perspective

The diagnosis of NMOSD came as a life-altering shock for our family, leaving us feeling as though the entire world had collapsed. Yet, in contrast to our despair, my daughter met the situation with laughter and hope, as she always does. Despite the uncertainty about what the future would hold, we clung to the hope of a miracle.

When we first heard about the treatment with corticosteroids, specifically methylprednisolone, we knew very little about it. We had only heard that it could work wonders in some cases but might have negative effects in others. Still, we placed our hopes in this treatment and took every step to support it, including maintaining the appropriate diet for our daughter. We understood that the corticosteroids were primarily for managing acute relapses, but for long-term control, she needed an immunosuppressant; mycophenolate was recommended to prevent further relapses and help her lead as normal a life as possible. We followed the doctor’s instructions and adhered to the medication guidelines, hoping for stability so we could return home and reclaim a normal life.

Unfortunately, the initial treatment did not provide the results we had hoped for. The disease continued to progress; the condition required a stronger intervention. Rituximab was recommended, a medication that required additional investigations, extensive monitoring, and administration in the intensive care unit due to its potentially serious side effects. Hearing about the risks caused me significant anxiety.

We followed the prescribed course, completing the first and second doses of rituximab without major issues. However, before the third dose, severe adverse reactions appeared. It was one of the most frightening moments of my life, as I feared losing my daughter. Despite the fear, I believed in her inner strength, which she had always shown, and held onto the hope that she would not give up.

Over time, rituximab proved effective in preventing relapses and helping regain some normalcy in her life. It gave her a new chance—a life that, while different, allowed her to enjoy being a child again. Ever resilient, she embraced this new life with determination, diligently following her treatment plan and looking forward to the future with optimism.

## Figures and Tables

**Table 1:** CD19 monitoring after rituximab infusion.

Dose	CD19 Monitoring
Pre-Dose I	6.1%
1 month after dose I	0.2%
6 months after dose I	0.5%
12 months after dose I	8.7%
18 months after dose I	7%
24 months after dose I	0%

## Data Availability

The original data presented in the study are included in the article.
